# “Decompression illness” on extracorporeal membrane oxygenation

**DOI:** 10.1186/s13019-024-02815-7

**Published:** 2024-06-01

**Authors:** Jiannan Hu, Huijing zhao, BingBing Bian, Renfei San, Peng Yang, Yongpo Jiang

**Affiliations:** 1 Department of Critical Care, Taizhou Hospital of Zhejiang Province affiliated to Wenzhou Medical University, No.150,XiMen Street, Taizhou, 317000 China; 2 Department of Emergency Medicine, Taizhou Hospital of Zhejiang Province affiliated to Wenzhou Medical University, Taizhou, 317000 China; 3 Department of Public Research Platform, Taizhou Hospital of Zhejiang Province affiliated to Wenzhou Medical University, Taizhou, 317000 China

**Keywords:** Extracorporeal membrane oxygenation, Air, Gas desorption, Decompression sickness, Complication

## Abstract

**Background:**

Extracorporeal membrane oxygenation (ECMO) is increasingly being used for critically ill patients with cardiopulmonary failure. Air in the ECMO circuit is an emergency, a rare but fatal complication.

**Case presentation:**

We introduce a case of a 76-year-old female who suffered from cardiac arrest complicated with severe trauma and was administered veno-arterial extracorporeal membrane oxygenation. In managing the patient with ECMO, air entered the ECMO circuit, which had not come out nor was folded or broken. Although the ECMO flow was quickly re-established, the patient died 6 h after initiating ECMO therapy.

**Conclusions:**

In this case report, the reason for the complication is drainage insufficiency. This phenomenon is similar to decompression sickness. Understanding this complication is very helpful for educating the ECMO team for preventing this rare but devastating complication of fatal decompression sickness in patients on ECMO.

## Background

Extracorporeal membrane oxygenation (ECMO) has received particular attention during the coronavirus disease 2019 (COVID-19) pandemic [1]. ECMO is the ultimate treatment strategy for patients with acute respiratory distress syndrome (ARDS) [2]. ECMO is a high-risk rescue technique, and the complications of ECMO are often fatal [3]. Renal failure, infection, and hemorrhage are common complications of ECMO [4]. Air in the ECMO circuit is an emergency, and a rapid response is needed [5]. The incidence of air in the ECMO circuit has been reported to be 1.4–4.6% [6]. We introduce a new case of air in the ECMO circuit, which is similar to decompression sickness. Decompression sickness is induced by a decrease in pressure that leads to the supersaturation of the tissue with dissolved gas and the subsequent evolution of gas bubbles [7]. We searched the literature and summarized various causes of air entering the ECMO circuit. Written informed consent was obtained from the patient’s family for the publication of this manuscript and any accompanying images. This manuscript adheres to the applicable EQUATOR guideline.

## Case presentation

**Fig. 1:**
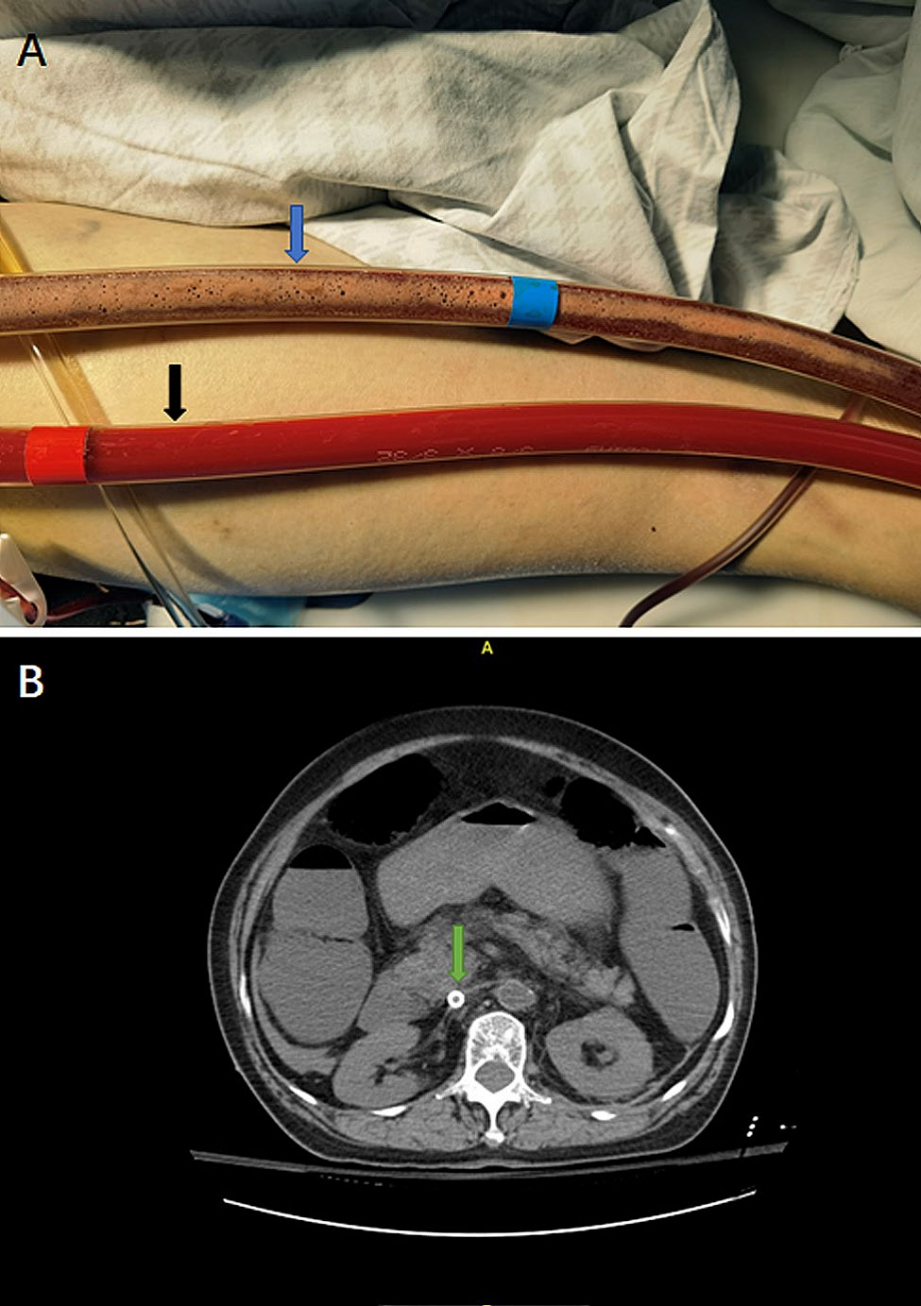
(**A**)The bright red tube is the arterial line of the ECMO (black arrow); the dark tube with many bubbles is the venous line of the ECMO (blue arrow). There are many small bubbles were formed and attached to the venous line of the ECMO. (**B**) Abdominal computed tomography showed that the inferior vena cava had collapsed completely(green arrow)

A 76-year-old female patient with a history of hypertension was admitted to the emergency department for cardiac arrest caused by severe trauma from a car accident. Continuous cardiac compressions for 30 min, the patient did not recover autonomous rhythm. Veno-arterial extracorporeal membrane oxygenation (ECMO) treatment was administered immediately for cardiac arrest. Percutaneous IVC-FA ECMO (21 F/45 cm single-stage venous cannulation: 96370-023; Medtronic, Inc. Minneapolis, MN, USA, and 15 F/18 cm arterial cannulation: 96570-015; Medtronic, MN, USA) was initiated for cardiac arrest. The ECMO initial settings were a pump speed of 3000 rpm and blood flow was only maintained from 0.3 to 0.6 L/min. We tried to decrease the rotating speed of ECMO to optimize ECMO traffic. After approximately 10 min, many small bubbles were formed and attached to the venous line of the ECMO (Fig. 1A). After inspection, the tube had not come out nor was folded or broken. The gas only stayed at the venous line and did not enter the pump, oxygenator, or arterial line. Immediately, both ends of the bubbled pipe were clamped and reconnected after the pipe was cut and the air bubbles were removed. The ECMO flow was then gradually increased from 1.8 to 2.0 L/min after fluid resuscitation. Abdominal computed tomography reveals collapse of the inferior vena cava, but no vascular damage or air is observed(Fig. 1B). Unfortunately, the patient died 6 h after initiating ECMO therapy.

## Discussion and conclusions

**Fig. 2:**
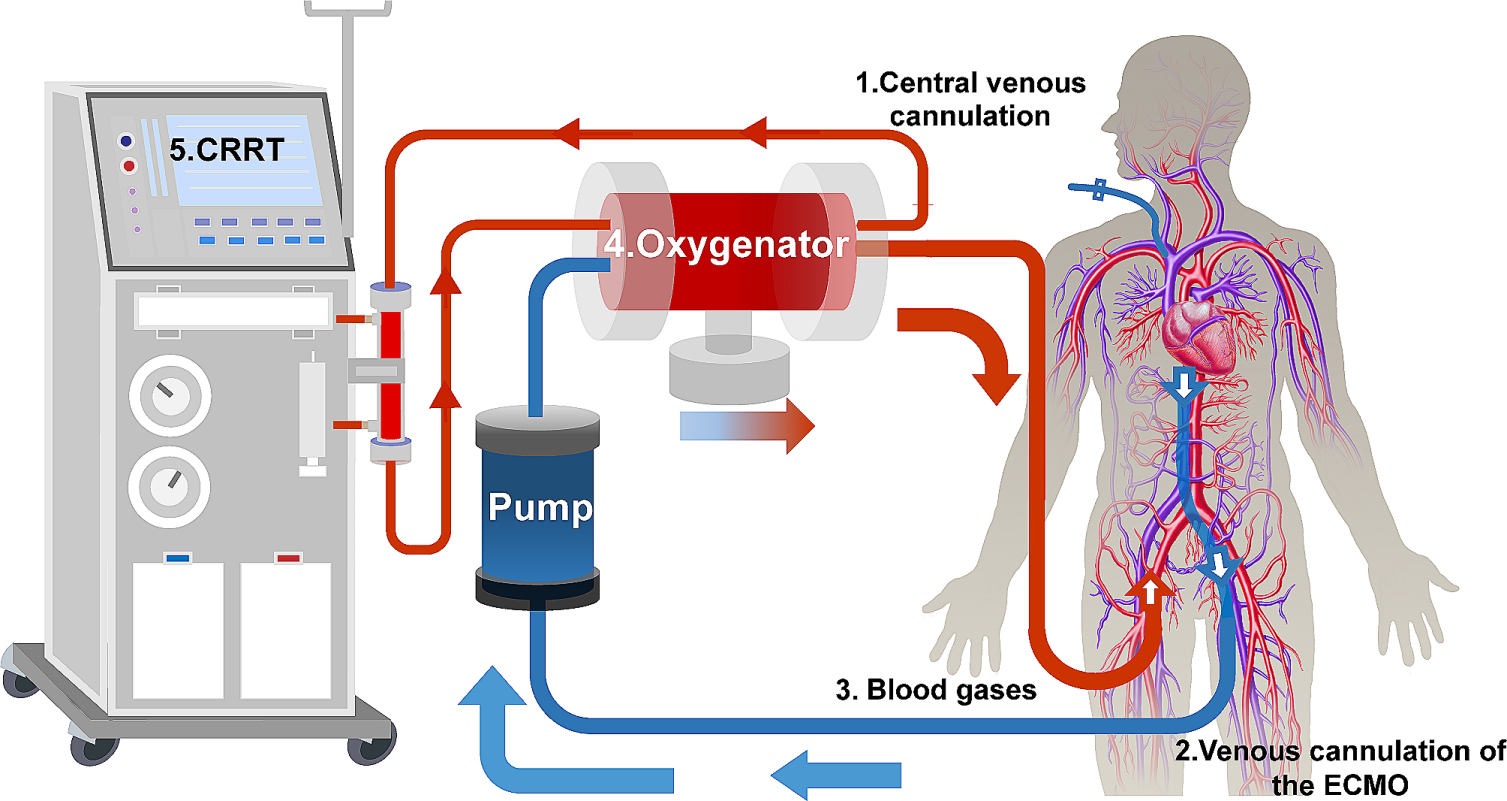
ECMO in combination with CRRT, The location of common pipeline inlet gas.(1. Central venous cannulation 2. venous cannulation of the ECMO 3. Blood gases 4. Oxygenator 5. CRRT )

Extracorporeal membrane oxygenation(ECMO) is the last treatment option for critically ill patients suffering from respiratory and circulatory failure or even cardiac arrest [8]. Air embolism in the ECMO circuit is a lethal and iatrogenic complication. Several factors contribute to gas entering the ECMO circuit, which is listed as follows: (1) gas accidentally transits from deep veins to ECMO pipelines during venipuncture. (2) Gas enters into the side hole due to the pressure difference when adjusting or withdrawing the side hole because the ECMO venous line takes off, leading to severe gas embolism. (3) gas enters the oxygenator. There are three channels for air, blood, and water in the oxygenator. The oxygenator is under positive pressure when the pump works and under negative pressure when the pump temporarily runs, which may allow gas to enter the membrane for a prolonged period; or (4) the extension of continuous renal replacement therapy(CRRT) pipelines, including many joints and tees, increases the possibility of gas entering pipelines when patients are treated with ECMO in combination with CRRT (Fig. 2). There are, of course, patient-related reasons for air intake in the ECMO circuit, including bronchovenous fistula and other vascular injuries. It is known that even after reconnecting the ECMO circuit, gas will continue to appear in the circuit if these underlying causes are not addressed. In this case, the cause of the air intake in the ECMO circuit can essentially be ruled out as being patient-related. Simultaneously, we observed that the gas primarily clung to the tubing walls, rather than entering the oxygenator with the bloodstream. This phenomenon further substantiates that the gas originates from dissolution processes within blood and other liquids. Should the presence of gas result from tube detachment, vascular rupture, or similar incidents, it would swiftly reach the oxygenator. We present the case of air bubbles appearing in the pipelines during ECMO therapy. In this case, gas that has quickly dissolved in the blood turns into bubbles, which is different from gas entering the pipelines. Insufficient venous return is caused by cardiac arrest and trauma (hypovolemia), whereas high pump speed relative to inflow resistance and blood volume results in excessively negative drainage pressure. When the ECMO flow rate is not smooth, a large negative pressure forms in the venous pipeline of the ECMO machine. The reason may be due to a contradiction between insufficient venous return and excessively negative drainage pressure. This occurrence is similar to that of decompression sickness [7].

Excessive negative pressure can cause ‘gas desorption’ of dissolved gases, such as carbon dioxide and oxygen, from the blood. Generally, maintaining a negative pressure of -200 mmHg in the venous segment of the ECMO circuit is deemed safe. However, surpassing a pressure differential equivalent to one atmosphere (760 mmHg) markedly elevates the risk of gas desorption. Air bubbles rarely occur in the ECMO circuit when it is well sealed. When the pump speed and blood flow are extremely mismatched, it is vital for us to pay attention to ECMO decompression illness caused by drainage insufficiency [9]. Air intake in this pipeline is different from the above four conditions. In either case, gas entering ECMO pipelines is lethal. With effective management, air in the circuit could be avoided during ECMO therapy. If this happens, we need to immediately clip the ECMO loop and check the reasons. Circuit change or deairing using the backflush technique could be used to re-establish ECMO flow [6]. Air in circuits leads to deleterious consequences, such as cerebral air embolism[5]. Decompression illness during extracorporeal membrane oxygenation should never be overemphasized, especially during special procedures.

## Data Availability

No datasets were generated or analysed during the current study.

## Abbreviations

ECMO: Extracorporeal membrane oxygenation
CRRT: Continuous Renal Replacement Therapy
